# Supplementary material for: split-intein Gal4 provides intersectional genetic labeling that is fully repressible by Gal80
Source: bioRxiv. 2023 Mar 24:2023.03.24.534001. Preprint. [Version 1] doi: 10.1101/2023.03.24.534001 (PMC10055387; doi:10.1101/2023.03.24.534001)
Supplement: Supplement 1 — Figure S1. Pilot characterization of split-intein Gal4 and Nanotag split-Gal4 in S2R+ cells. Plasmids encoding Gal4, split-Gal4, split-intein Gal4, or Nanotag split-Gal4, each driven by a constitutively expressed Actin promoter, were transiently transfected into S2R+ cells, either with or without co-transfection of the Gal80 repressor. Figure S2. In the absence of tub-Gal80ts, split-intein Gal4 functions at 18°C, 25°C, and 29°C. Larvae were reared at the indicated temperature, and live-imaged at the L3 stage under identical imaging conditions. As with wildtype Gal4 (27), split-intein Gal4 activity is strongest at 29°C and decreases as the rearing temperature is lowered. The right-most panel indicates negative control, with inset showing an overexposed image to indicate the presence of larva. Figure S3. Enhancer-driven expression of the split-intein system. (A) Gateway LR cloning strategy for cloning split-intein Gal4 components downstream of an enhancer-of-interest. This protocol follows closely the workflow used to generate the split-Gal4 “VT” collection based on 2–3kb enhancer fragments that drive expression in the fly nervous system. (B) Proof of principle for the VT024642 enhancer fragment driving Gal4N-int in the adult ISCs. Figure S4. Characterization of the split-intein GeneSwitch system using multiple drivers. Split-intein GeneSwitch expression in the adult ISCs using either esg (A) or Dl (B) is non-leaky, and is only observed in the presence of RU. (C) split-intein GeneSwitch expression in enterocytes throughout the adult midgut using Myo1A. In the absence of RU (left), expression is observed in a portion of the midgut, whereas RU drives the predicted expression throughout the gut. (D) When split-intein GeneSwitch is expressed ubiquitously using the tub promoter, non-RU-dependent expression is visible in portions of the hindgut. actin:GeneSwitch (original GeneSwitch) is expressed at lower levels than the tub promoter, and does not display this same leakines [file media-1.pdf]

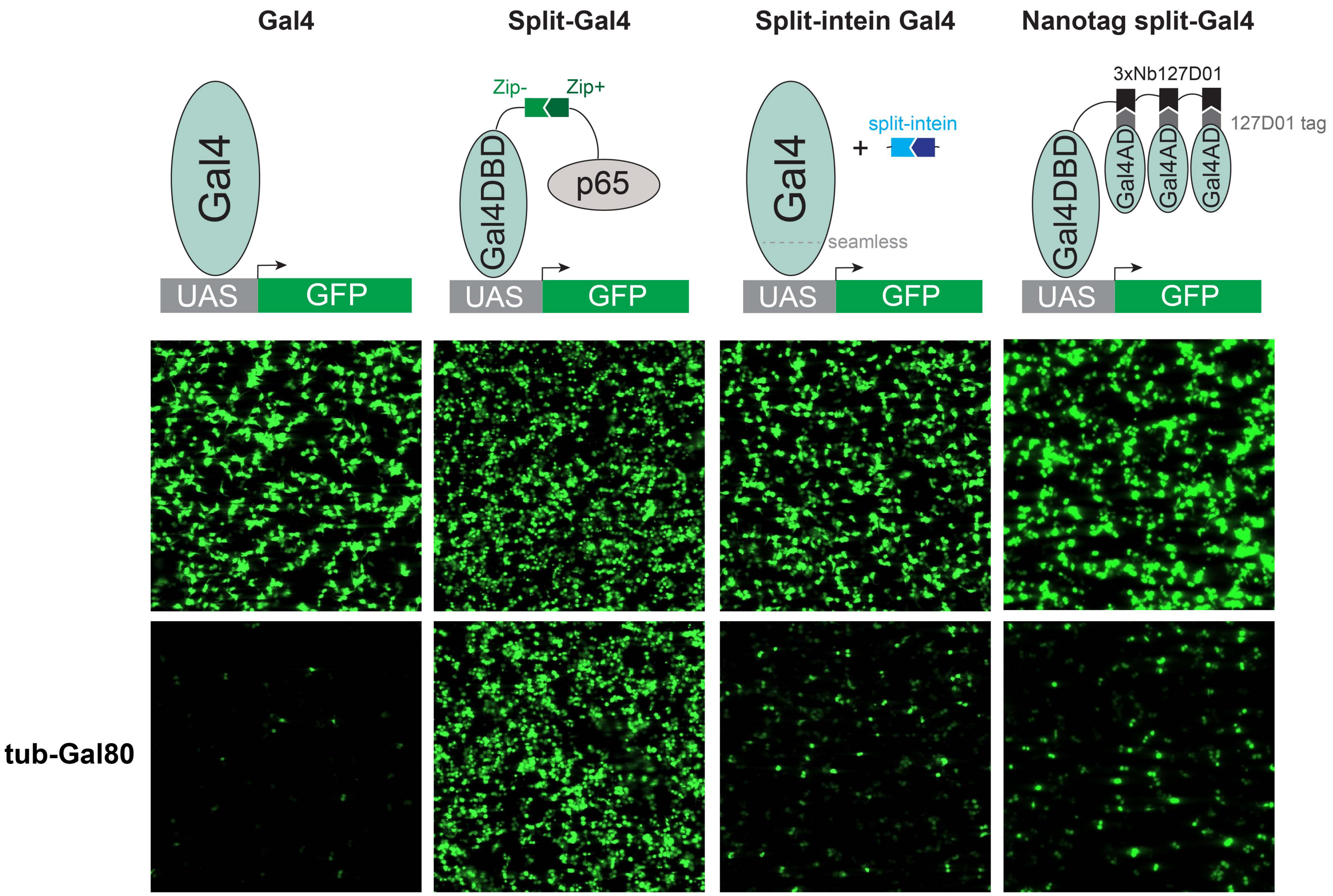
Gal4
Split-Gal4
Split-intein Gal4
Nanotag split-Gal4
Gal4
UAS
GFP
Zip-
Zip+
Gal4DBD
p65
UAS
GFP
Gal4
+
split-intein
seamless
UAS
GFP
3xNb127D01
127D01 tag
Gal4DBD
Gal4AD
Gal4AD
Gal4AD
UAS
GFP
tub-Gal80

Figure S1

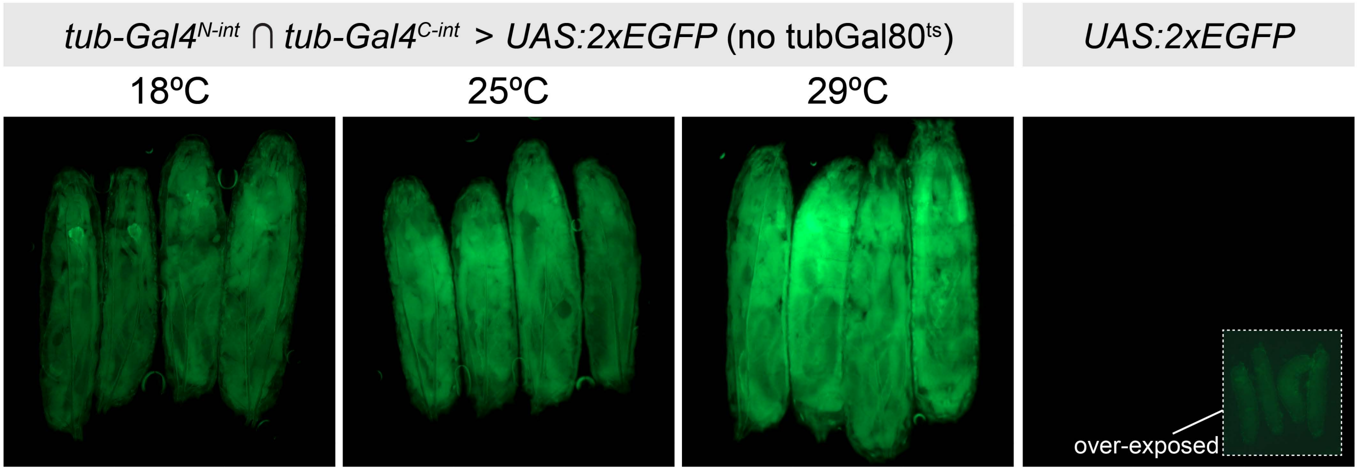
tub-Gal4N-int ∩ tub-Gal4C-int > UAS:2xEGFP (no tubGal80ts)
UAS:2xEGFP
18ºC
25ºC
29ºC
over-exposed

Figure S2

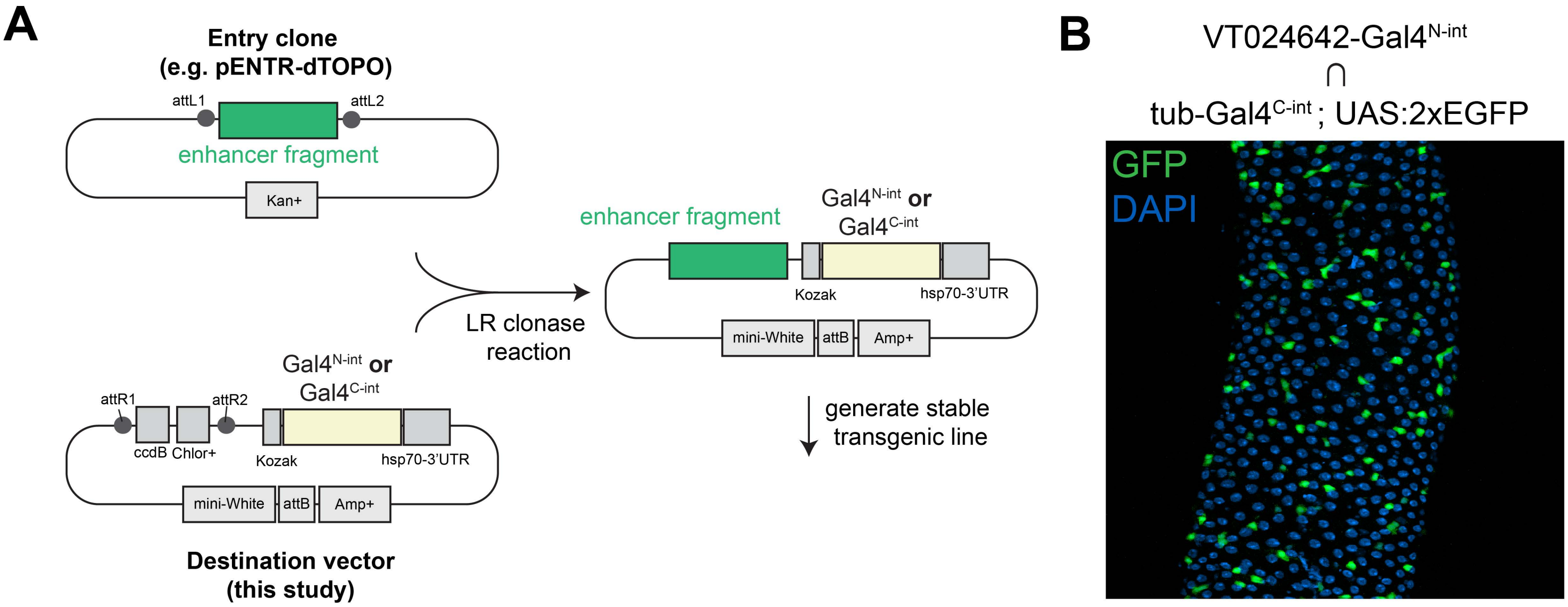
A
Entry clone
(e.g. pENTR-dTOPO)
attL1
attL2
enhancer fragment
Kan+
LR clonase
reaction
enhancer fragment
Gal4N-int or
Gal4C-int
Kozak
hsp70-3'UTR
mini-White
attB
Amp+
generate stable
transgenic line
Gal4N-int or
Gal4C-int
attR1
attR2
ccdB
Chlor+
Kozak
hsp70-3'UTR
mini-White
attB
Amp+
Destination vector
(this study)
B
VT024642-Gal4N-int
∩
tub-Gal4C-int; UAS:2xEGFP
GFP
DAPI

Figure S3

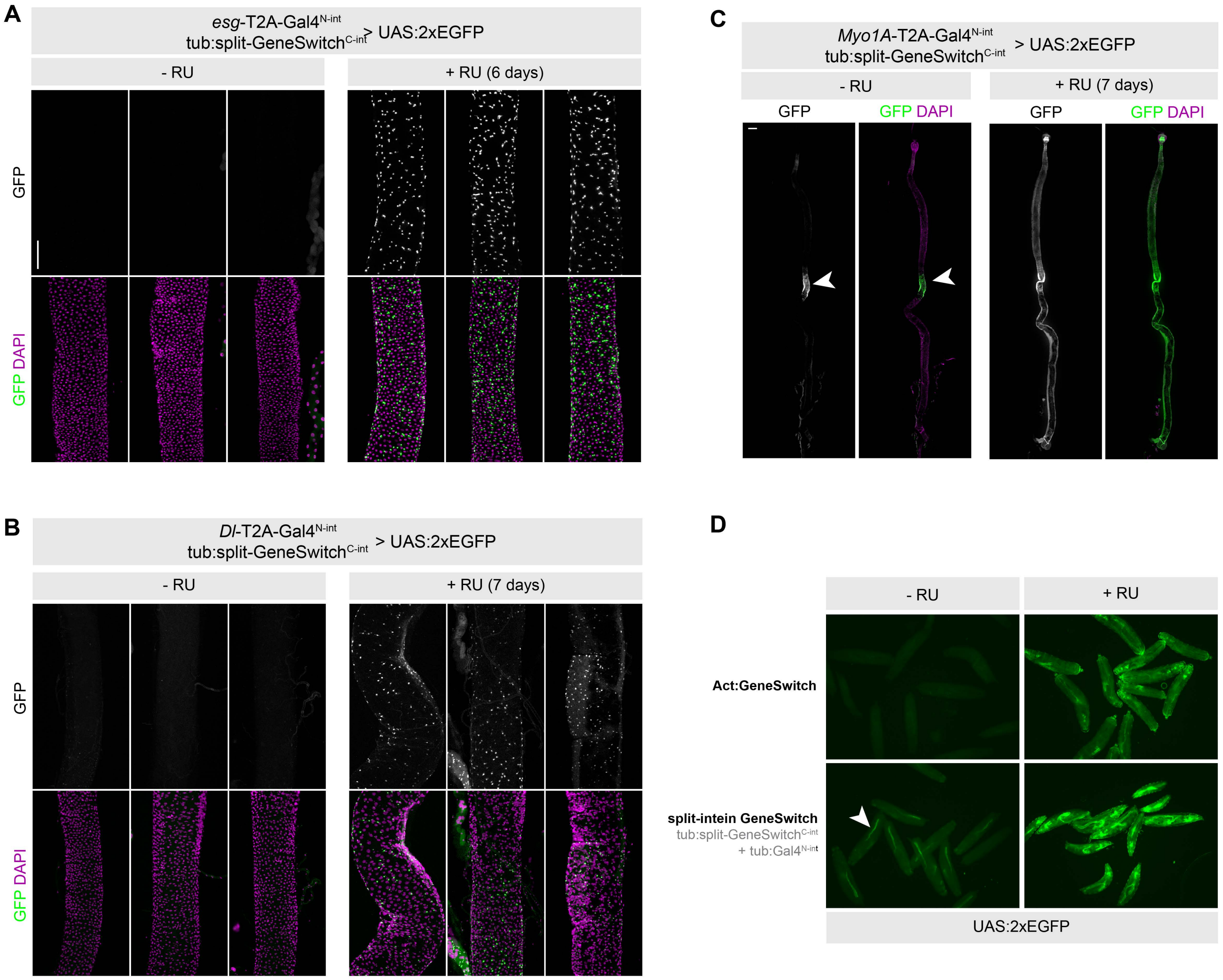
A
esg-T2A-Gal4N-int
tub:split-GeneSwitchC-int > UAS:2xEGFP
- RU
+ RU (6 days)
GFP
GFP DAPI
B
Dl-T2A-Gal4N-int
tub:split-GeneSwitchC-int > UAS:2xEGFP
- RU
+ RU (7 days)
GFP
GFP DAPI
C
Myo1A-T2A-Gal4N-int
tub:split-GeneSwitchC-int > UAS:2xEGFP
- RU
+ RU (7 days)
GFP
GFP DAPI
D
- RU
+ RU
Act:GeneSwitch
split-intein GeneSwitch
tub:split-GeneSwitchC-int
+ tub:Gal4N-int
UAS:2xEGFP

Figure S4

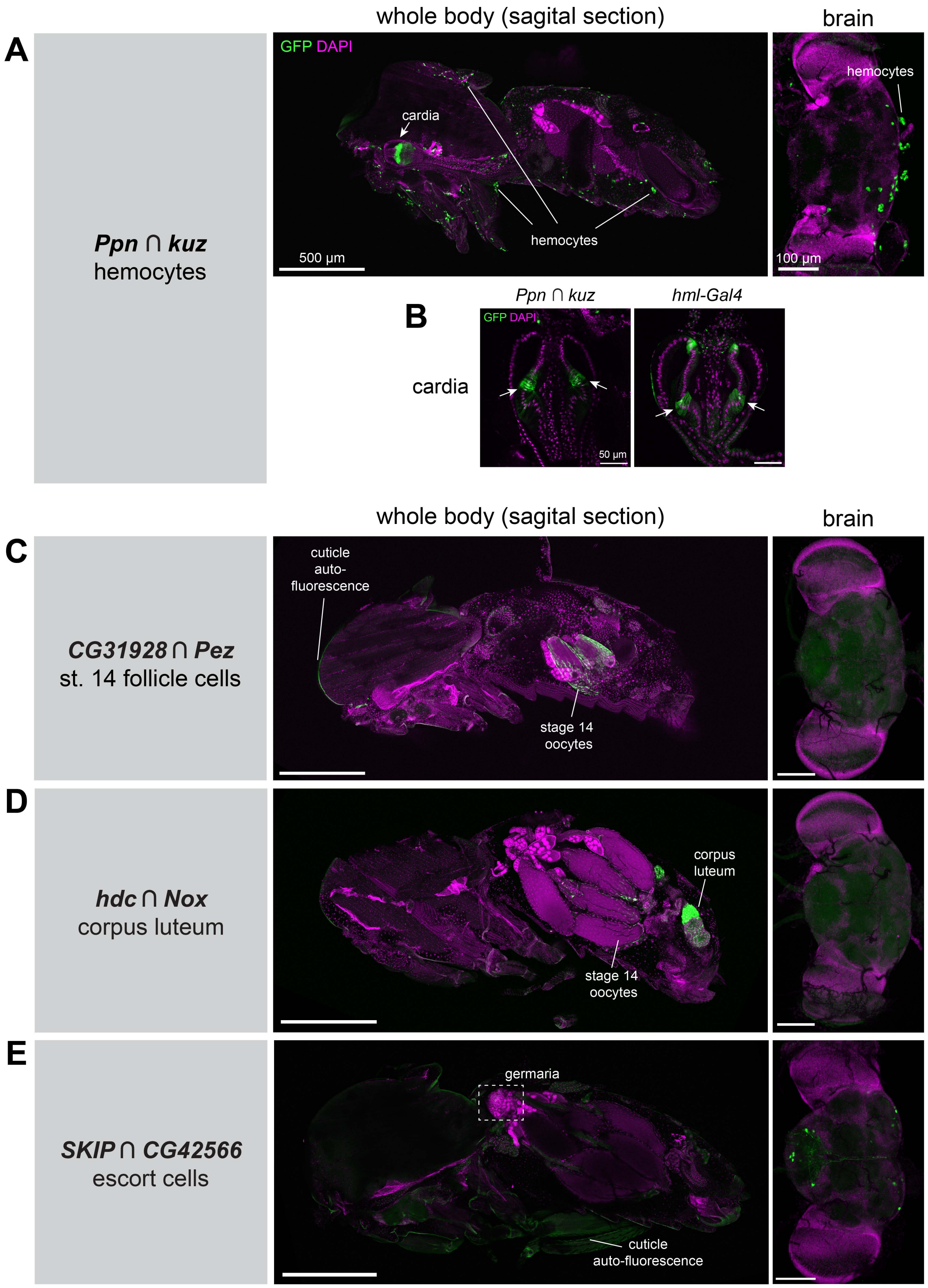
A
Ppn ∩ kuz
hemocytes
whole body (sagital section)
brain
GFP DAPI
cardia
hemocytes
hemocytes
500 µm
100 µm
B
Ppn ∩ kuz
hml-Gal4
cardia
GFP DAPI
50 µm
C
CG31928 ∩ Pez
st. 14 follicle cells
whole body (sagital section)
brain
cuticle
auto-
fluorescence
stage 14
oocytes
D
hdc ∩ Nox
corpus luteum
corpus
luteum
stage 14
oocytes
E
SKIP ∩ CG42566
escort cells
germaria
cuticle
auto-fluorescence

Figure S5

Purpose (Transgenesis method) | **Plasmid map** | **Cloning strategy**

pHD-T2A-split-inteinGal4

Purpose: Long homology arms (CRISPR knock-in)

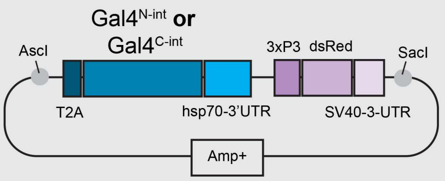

1. Digest with AscI + SacI
2. PCR amplify homology arms w/ overhangs
3. Gibson cloning

Reference: *Bosch et al. 2019*

pDropIn-split-inteinGal4

Purpose: "Drop-in" cloning (CRISPR knock-in)

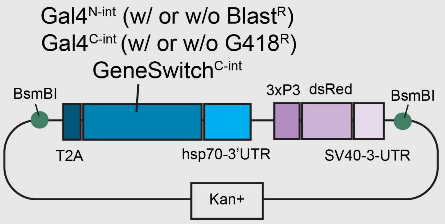

1. Release insert with BsmBI digest
2. Ligate into digested drop-in cassette

Reference: *Kanca et al. 2019*

pBP-split-inteinGal4-destination

Purpose: Enhancer driven (phiC31 integrase transgenesis)

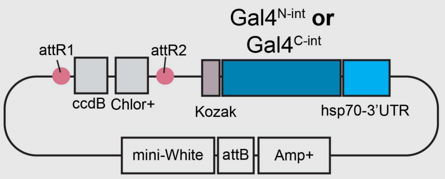

1. Clone enhancer into Gateway donor vector
2. Perform LR Clonase reaction

Reference: *Pfeiffer et al. 2008*

Figure S6
