# Supplementary material for: split-intein Gal4 provides intersectional genetic labeling that is fully repressible by Gal80
Source: bioRxiv. 2023 Mar 24:2023.03.24.534001. Preprint. [Version 1] doi: 10.1101/2023.03.24.534001 (PMC10055387; doi:10.1101/2023.03.24.534001)
Supplement: Supplement 2 [file NIHPP2023.03.24.534001v1-supplement-2.pdf]

bioRxiv preprint doi: https://doi.org/10.1101/2023.03.24.534001; this version posted March 24, 2023. The copyright holder for this preprint (which was not certified by peer review) is the author/funder, who has granted bioRxiv a license to display the preprint in perpetuity. It is made available under aCC-BY 4.0 International license.

Table S1: Transgenic Drosophila lines generated in this study

Line name	Genotype*	sgRNA for knock-in
esg-T2A-Gal4[N-int]	*w ; TI{T2A-Gal4-N-int}esg / CyO*	CTCCACCAACATGTCTTCCA
Myo1A-T2A-Gal4[N-int]	*w ; TI{T2A-Gal4-N-int}Myo1A / CyO*	CCATGGACACTTGGTCGAGG
Delta-T2A-Gal4[C-int]	*w ;; TI{T2A-Gal4-C-int}Dl / TM6b*	TCGCTGCTGCAGCGGGGAGT
esg-T2A-Gal4DBD	*w ; TI{T2A-Gal4DBD}esg / CyO*	CTCCACCAACATGTCTTCCA
Myo1A-T2A-Gal4DBD	*w ; TI{T2A-Gal4DBD}Myo1A / CyO*	CCATGGACACTTGGTCGAGG
tub-Gal4[N-int]	*w ; P{y[+t7.7]w[+mC]alphaTubulin48B-Gal4-N-int}attP40*	N/A
tub-Gal4[C-int]	*w ; P{y[+t7.7]w[+mC]alphaTubulin48B-Gal4-C-int}attP40*	N/A
tub-GeneSwitch[C-int]	*w ; PI{y[+t7.7]w[+mC]alphaTubulin48B-GeneSwitch-C-int}attP40*	N/A
VT024642-Gal4[N-int]	*w ; P{y[+t7.7]w[+mC]VT024642-Gal4-N-int}attP40*	N/A
Peritrophin-15a-T2A-Gal4[N-int]	*w ; TI{T2A-Gal4-N-int}Peritrophin-15a / CyO*	TTCGTGGCGCTCCTAAGCAC
CG4830-T2A-Gal4[C-int]	*w ;; TI{T2A-Gal4-C-int}CG4830 / TM6b*	GTTACCTTGCAGCTACGATG
CG43774-T2A-Gal4[N-int]	*w ; TI{T2A-Gal4-N-int}CG43774 / CyO*	TACGCGAATCCATTGGCTTG
thetaTry-T2A-Gal4[C-int]	*w ; TI{T2A-Gal4-C-int}thetaTry / CyO*	GGCACAGTCGGGGTCTCCAA
LManV-T2A-Gal4[N-int]	*w ; TI{T2A-Gal4-N-int}LManV / CyO*	AAGTCAGCCCAAGCTGTTTG
ninaD-T2A-Gal4[C-int]	*w ; TI{T2A-Gal4-C-int}ninaD / CyO*	CGGCCTGGGAACCTTTTTCG
Ppn-T2A-Gal4[N-int]	*w ;; TI{T2A-Gal4-N-int}Ppn / TM6b*	TGGGGCGAACATGTACTTGC
kuz-T2A-Gal4[C-int]	*w ; TI{T2A-Gal4-C-int}kuz / CyO*	ATAGTTGAGTGTTTCATAGT
CG31928-T2A-Gal4[N-int]	*w ; TI{T2A-Gal4-N-int}CG31928 / CyO*	ACCGAGTACTATGTTACGGC
Pez-T2A-Gal4[C-int]	*w ; TI{T2A-Gal4-C-int}Pez / CyO*	GTTCGCCACTACGTGACCAC
hdc-T2A-Gal4[N-int]	*w ;; TI{T2A-Gal4-N-int}hdc / TM6b*	CCTGAACGAGCTCTCCCTGG

bioRxiv preprint doi: https://doi.org/10.1101/2023.03.24.534001; this version posted March 24, 2023. The copyright holder for this preprint (which was not certified by peer review) is the author/funder, who has granted bioRxiv a license to display the preprint in perpetuity. It is made available under aCC-BY 4.0 International license.

Nox-T2A-Gal4[C-int]	*w ; TI{T2A-Gal4-C-int}Nox / CyO*	AGGGCGTGGCGGGAATCTCC
SKIP-T2A-Gal4[N-int]	*w ;; TI{T2A-Gal4-N-int}SKIP / TM6b*	AGCTCTCGGCGTATTGGGCC
CG42566-T2A-Gal4[C-int]	*w ; TI{T2A-Gal4-C-int}CG42566 / CyO*	TGGCCCGGAACCGTGCATCG
CG13321-T2A-Gal4[N-int] + CG6484-T2A-Gal4[C-int] ; UAS:2xEGFP	*w ; TI{T2A-Gal4-N-int}CG13321, TI{T2A-Gal4-C-int}CG6484 / CyO ; UAS:2xEGFP / TM6b*	CCAGCTAGGATGGCTCCTGG, ATTGTGTCGGGCGTGCTGTA

** May be segregating nos:Cas9 on a separate chromosome*
